# Health, harm reduction, and social service providers’ perspectives on the appropriateness and feasibility of peer distribution of HIV self-test kits among people who use drugs

**DOI:** 10.1186/s12954-024-00950-x

**Published:** 2024-02-04

**Authors:** Angela R. Bazzi, Chad J. Valasek, Tara Stamos-Buesig, William H. Eger, Alicia Harvey-Vera, Carlos F. Vera, Jennifer L. Syvertsen, Erik D. Storholm, Tyler S. Bartholomew, Hansel E. Tookes, Steffanie A. Strathdee, Heather A. Pines

**Affiliations:** 1 Herbert Wertheim School of Public Health, University of California, San Diego, 9500 Gilman Drive, MTF 265E (Mail Code 0725), La Jolla, CA 92161 USA; 2 School of Public Health, Boston University, Boston, MA USA; 3 School of Medicine, University of California, San Diego, La Jolla, CA USA; 4 OnPoint, Harm Reduction Coalition of San Diego, San Diego, CA USA; 5 School of Social Work, San Diego State University, San Diego, CA USA; 6 Department of Anthropology, University of California, Riverside, Riverside, CA USA; 7 School of Public Health, San Diego State University, San Diego, USA; 8 University of Miami Miller School of Medicine, Miami, FL USA

**Keywords:** People who inject drugs, HIV self-testing, Secondary distribution, Social networks, HIV prevention, Harm reduction

## Abstract

**Background:**

People who use drugs (PWUD) experience elevated HIV risk and numerous barriers to facility-based HIV testing. HIV self-testing (HIVST) could circumvent many of those barriers and is acceptable among PWUD, yet HIVST implementation for PWUD is limited. Service providers’ perspectives on specific HIVST delivery strategies could help increase availability for PWUD.

**Methods:**

From April–November 2021, we interviewed 16 health, harm reduction, and social service providers working with PWUD in San Diego, CA. Interviews and rapid thematic analysis explored perspectives on HIVST’s utility and appropriateness, as well as the feasibility of and anticipated challenges with specific HIVST delivery strategies, including peer or secondary distribution.

**Results:**

Participants viewed HIV as a significant threat to PWUD health and confirmed the presence of numerous barriers to local facility-based HIV testing. Participants viewed HIVST as a promising and potentially empowering solution. Based on community familiarity with secondary distribution of harm reduction supplies (i.e., naloxone) and information, participants viewed secondary distribution of HIVST kits as an appropriate and feasible strategy for increasing the reach of HIVST, but also described potential barriers (e.g., engaging socially disconnected individuals, ensuring linkages to services following HIVST) and provided suggestions for alternative HIVST kit delivery models (e.g., harm reduction vending machines).

**Conclusions:**

Service providers viewed secondary distribution of HIVST kits among PWUD as promising, appropriate, and feasible, yet specialized efforts may be needed to reach the most marginalized individuals and ensure consistent provision of educational information and referral supports that maximize the impact of this approach.

## Background

People who use and inject drugs (hereafter, PWUD) experience elevated HIV risk in the context of the ongoing opioid and polysubstance use epidemics in the United States [1]. Increased exposure to HIV is likely a result of widespread fentanyl use, which increases injection frequency and syringe sharing, and stimulant use associated with sexual transmission of HIV [2]. Recent HIV outbreaks in diverse U.S. regions have caused renewed attention to HIV transmission and prevention and treatment needs among PWUD [3].

HIV testing, a critical first step to entering the HIV prevention and treatment cascades and is recommended at least annually for people who inject drugs in the United States [4]. However, only 55% of 2018 National HIV Behavioral Surveillance Survey participants representing this population reported being tested for HIV in the past 12 months [5]. Despite frequent sexual and injection-related exposures to HIV, social and structural barriers to healthcare utilization (e.g., stigma, homelessness, transportation challenges, criminal justice involvement) likely reduce rates of HIV testing observed among PWUD [6–12]. For those who do access HIV testing, similar barriers limit engagement in subsequent HIV prevention and treatment services, contributing to low levels of pre-exposure prophylaxis (PrEP) uptake, late HIV diagnosis, suboptimal antiretroviral (ART) initiation and adherence, lack of viral suppression and ongoing transmission, and unmet health and social needs [13–16].

HIV self-testing (HIVST) could help increase HIV testing among PWUD by circumventing many of the barriers to traditional, facility-based HIV testing [17, 18]. HIVST enables convenient, potentially discreet testing that is effective in increasing diagnosis in diverse populations [17, 19–22]. Although a small number of studies have found HIVST to be acceptable among PWUD, particularly when coupled with harm reduction services [23–25], the feasibility and ultimate impact of HIVST for PWUD will require the identification of effective delivery strategies. While mail and facility-based distribution approaches have increased access to HIVST for other populations, these approaches may be less beneficial for engaging the most socially and structurally marginalized PWUD experiencing homelessness and other barriers described above. Social network-based delivery (i.e., peer or secondary distribution) of HIVST kits has helped increase the diffusion of HIVST among other populations with limited healthcare access, including sex workers’ partners and network members of men who have sex with men who report never or infrequent HIV testing [20, 26–29]. To investigate whether and how to best implement peer or secondary distribution of HIVST kits for PWUD, we explored health, harm reduction, and social service providers’ perspectives on the appropriateness and feasibility of this approach to inform future HIVST intervention and implementation efforts for this population.

## Methods

### Study design and sample

From April to November 2021, we conducted qualitative interviews with health, harm reduction, and social service providers who were ≥ 18 years of age and reported working professionally with PWUD in San Diego County, CA, an Ending the HIV Epidemic (EHE) priority jurisdiction [3] where HIV transmission has been linked to international drug trafficking of heroin, fentanyl, and methamphetamine [30–32]. Although the primary objective of the original research study was to explore service providers’ perspectives on delivering long-acting injectable PrEP to PWUD [33], barriers to HIV testing that emerged in early interviews led us to develop new questions exploring the delivery of HIVST kits to PWUD as a potential strategy to overcoming those barriers. We recruited individuals through our professional networks, using input from members of a local Community and Scientific Advisory Board [34] and enrolled participants (i.e., snowball sampling) [35]. Individuals provided verbal informed consent and received $50 compensation for their time. The institutional review board of the University of California, San Diego reviewed and approved all study procedures and granted a waiver of documentation of consent.

### Data collection

An experienced qualitative researcher (CJV) conducted interviews using a semi-structured interview guide designed to explore service providers’ perspectives on the implementation of long-acting injectable PrEP among PWUD [33], more general barriers to HIV testing and prevention services in the region, possible strategies to overcome identified barriers, including HIVST, and the appropriateness and feasibility of specific HIVST kit delivery strategies, including peer or secondary distribution within the social networks of PWUD. Interviews were conducted in-person or virtually, lasted 45 min on average, and were audio-recorded and professionally transcribed verbatim following a structured protocol involving redacting potential identifiers to protect participant confidentiality [36].

### Data analysis

Following an iterative yet systematic rapid assessment process [37, 38], immediately following interviews, the interviewer recorded detailed notes using a structured template containing fields for deductive topics (based on interview guide questions) and emergent themes (identified through interviewer notes, regular team meetings, and the ongoing analytic process) [39]. An additional analyst (ARB) independently reviewed and summarized transcripts using this template and included illustrative quotes when available. Templates were then combined into a single matrix displaying data across participants and topics, enabling rapid, targeted thematic analysis involving a primarily deductive approach to summarizing perspectives on the appropriateness and feasibility of specific HIVST kit delivery strategies [40]. We employed several strategies to enhance the trustworthiness of our qualitative findings, including triangulation of findings with recent quantitative research on this topic [41], reflexivity and community engagement among our research team members, peer review and debriefing with individuals external to our team, and the inclusion of harm reduction providers and individuals with lived experience within our team [39].

## Results

### Sample characteristics and overview of key findings

Sixteen participants represented healthcare and behavioral health services (e.g., clinicians, counselors; n = 7), harm reduction and homeless outreach services (n = 5), and the public health and safety sectors (n = 4). Participants reported working professionally with PWUD for a median of 10.0 years (interquartile range: 5.3–16.5 years). Although we did not directly ask, five participants (31.3%) disclosed having personal lived experience with substance use and/or homelessness during their interviews. From our targeted thematic analysis, we identified three interrelated themes related to the appropriateness and feasibility of peer distribution of HIVST kits among PWUD, which we identified consistently across the professions represented in our sample: (1) multilevel barriers limit access to local facility-based HIV testing and prevention services; (2) peer distribution of HIVST kits is likely appropriate and feasible due to community familiarity with secondary distribution of prevention supplies and information; and (3) anticipated barriers to implementing this novel approach could require additional strategies or supports. These themes are described in the sections below, illustrated as appropriate with anonymized, representative quotes.

#### Multilevel barriers limit access to local facility-based HIV testing and prevention services

Overall, participants viewed HIV as a significant threat to PWUD health and identified multilevel barriers that they believed limited PWUD access to facility-based HIV testing and prevention services in San Diego County. First, they explained that many PWUD have low awareness of HIV testing and prevention services because promotional efforts have not been geared towards this population, as one clinical provider stated, *“[HIV testing] is [available] but [PWUD] just don't think about [it] and providers don’t offer it as part of the regular menu of services.”* Specific barriers to HIV prevention services (e.g., PrEP) also included low knowledge and comfort among providers, who one clinical provider described as not “*wanting to deal with this population or not being familiar with [PrEP] or feeling comfortable prescribing it.”* Participants also described limited integration of HIV and addiction treatment services, in which HIV testing and prevention are “*not even on the radar.”* Compounding this clinical barrier, some participants expressed concern that public health agencies and clinics had de-prioritized HIV testing and prevention services, particularly for PWUD.

Most participants also identified structural barriers to facility-based HIV testing and prevention services for PWUD (e.g., transportation, health insurance, criminalization of drug use, and homelessness), emphasizing that daily survival needs reduced interest in and ability to access these services for PWUD. As one harm reduction professional explained, *“As far as remembering to show up in three days [for your HIV test results]? A lot of people don’t even know what day it is. ‘I might not even be alive in three days’ is their attitude.”*

#### Peer distribution of HIVST kits is likely appropriate and feasible

When asked about peer distribution of HIVST kits, nearly all participants viewed this approach as appropriate and feasible, yet none were aware of any current efforts to promote HIVST access in this way. When reflecting on the appropriateness and feasibility of peer distribution of HIVST kits, participants often compared this approach to the secondary distribution of prevention supplies and information, with which local communities of PWUD and harm reduction providers were already familiar. One harm reduction professional explained that PWUD actively *“spread knowledge in their social groups”* and distribute syringes, naloxone, and other harm reduction supplies within a culture of support:

For a long time, [PWUD have been] passing out fentanyl test strips and showing each other how to use them. And if someone doesn't know how to use them, they’ll find someone else who does. People in the houseless community of [PWUD] practice mutual aid more than anyone; they pool their resources to help each other.

Some participants also pointed out that, like the general population, COVID-19 testing had increased PWUD familiarity with self-testing technologies, as *“[PWUD] were accepting [COVID-19 tests] and sharing them in their camps.”* An additional benefit of HIVST kits, which could be used in privacy and at individuals’ discretion, could be empowering, according to one clinical provider:

It would allow self-determination. It puts responsibility and agency in [people’s] hands; democratization of healthcare access. If you can only get tested by a doctor, it’s a barrier…you may not want to go, you may be scared, embarrassed…but those barriers [are] gone once you have the test in your hand [and] you [can] decide what to do with it and when.

#### Anticipated barriers to peer distribution of HIVST kits could require additional strategies or supports

Participants emphasized the importance of anticipating specific barriers to optimizing the reach of peer-based distribution of HIVST kits, including community hesitancy and *“pushback”* related to the introduction of new, unfamiliar technologies (e.g., fentanyl test strips, which *“not a lot of people wanted”* when first introduced). Several participants also commented on the *“extra layer of stigma”* surrounding HIV, as one clinician explained: *“[There’s] an extra level of concern if you're identified as living with HIV and you're in a community where you're all using [drugs] together, it may jeopardize [your] safety and increase the shame and stigma.”* A couple of participants, including one with lived experience with drug use and homelessness locally, also warned that it could be difficult for peer distribution methods to reach PWUD with low social connectedness and limited peer support and trust (i.e., the *“Have Nots,”* who, as compared to the *“Have’s,”* are more likely to be experiencing unsheltered homelessness and social isolation, could be missed by some or most forms of peer outreach, and would be the least likely to be aware of local HIV services they could access to following HIVST).

Participants also provided several specific strategies for overcoming potential challenges with peer distribution of HIVST kits. First, several expressed the importance of providing information on local HIV services *“on multiple levels”* and by leveraging word of mouth throughout the PWUD community. One healthcare provider practicing street medicine explained how community awareness had increased over time, thus increasing the demand for relatively recently-introduced harm reduction supplies in San Diego County: *“I'm starting to get more and more requests for naloxone and [fentanyl] test strips, which [is] exciting because people are taking precautions. So it might take time, but [HIVST kits] would probably catch on just like that.”* A harm reduction professional described partnering with syringe services programs (SSPs) as particularly beneficial because *“even the downtrodden, [those] without money, show up there,”* especially at mobile SSP locations (e.g., pop-up tents) offering harm reduction supplies, donated clothing or camping supplies, or small incentives for service engagement. Finally, other novel distribution approaches such as including HIVST kits (with information on local HIV services) in harm reduction vending machines came up in a couple of interviews, with participants representing harm reduction services suggesting that these or other efforts to deliver HIVST kits would benefit from community input specifically* “from people we're trying to [serve].”*

## Discussion

Given the persistence of HIV transmission among PWUD in the United States [1–3], innovative efforts are needed to increase rates of HIV testing and subsequent engagement in prevention and treatment services. HIV self-testing (HIVST), which can occur outside of standard healthcare facilities, could help increase HIV testing among PWUD if widespread distribution is achieved [17, 18]. Our study confirmed that harm reduction and other service providers working with this population across San Diego County viewed HIVST as a promising, potentially empowering HIV testing method for PWUD and perceived peer distribution of HIVST kits to be appropriate and feasible. Here, we highlight several considerations and future directions for HIVST research and delivery.

First, participants confirmed that peer distribution of naloxone, fentanyl test strips, and other harm reduction supplies and information is already taking place within local communities. Secondary distribution of harm reduction supplies (e.g., syringes, naloxone) within the networks of syringe services program (SSP) clients has been occurring for decades and is believed to help extend SSPs’ reach into marginalized communities of PWUD who do not directly access onsite prevention services [42–50]. The engagement of peers (i.e., “secondary exchangers”) in SSPs’ HIV prevention efforts could be particularly helpful in large, geographically dispersed regions, or for reaching some of SSPs’ most vulnerable participants, including those experiencing unsheltered homelessness, who may face the greatest barriers to accessing on-site HIV testing at SSPs. However, our participants expressed concerns about HIV-related stigma and reaching PWUD with low social connectedness (i.e., the *“Have Nots”*) using secondary distribution, and provided specific suggestions (e.g., harm reduction vending machines) [51] for reaching some sub-populations of PWUD.

Second, some participants in our sample questioned how HIV education and information on local services (including PrEP and HIV treatment) could be consistently provided through secondary distribution of HIVST kits, particularly for individuals experiencing higher levels of social isolation. Much of the research on secondary distribution among PWUD has focused on the direct delivery of tangible harm reduction supplies (e.g., syringes) [44–47, 50, 52]; less is known about peer distribution of educational information and referral support that should accompany HIV testing. Although our participants described the informal sharing of educational information among PWUD, formalized peer-driven social network interventions have successfully promoted HIV risk reduction among PWUD [53–56]. Recognizing that peer educator selection can be critical to the success of these intervention [57, 58], research is needed to identify the optimal types of information, communication strategies, and centrally located peers that could support the optimal delivery of HIVST kits and related information. Specific training on the correct usage of HIVST kits may be needed for secondary distributors, as international studies engaging PWUD in hepatitis C virus self-testing (HCVST) have found that some individuals request more assistance using test kits and interpreting results than others [59–61]. Thus, successful implementation of HIVST among PWUD may require additional education on correct use and some availability of assistance throughout the testing process.

Third, it is worth noting that HIV testing is only an initial step towards engagement in the HIV prevention and care continuums. The extent to which individuals are willing and able to disclose their test results to public health programs (for population-level surveillance) or follow through on referrals to services remains largely unknown. Indeed, in focus groups with at-risk populations in Kenya, including PWUD, participants expressed concerns about accessing pre- and post-test counseling services [62]. Similarly, studies on the acceptability of HCVST among PWUD in Kyrgyzstan and the United Kingdom found that many participants wanted HCVST delivered through harm reduction organizations that could directly provide pre- and post-test counseling services and supported referrals to care, particularly for positive results [63, 64]. Additional research on how to best link PWUD using HIVST kits to post-test counseling, HIV prevention (e.g., PrEP), and treatment services will be critical to harnessing the full potential of the secondary distribution of HIVST kits for PWUD. These counseling and linkage supports could be provided through mobile or fixed SSP sites or other venues frequently accessed by this population (e.g., opioid treatment programs). Digital interventions have been developed to support service linkage following HIVST [65], yet PWUD most vulnerable to HIV often lack consistent phone and Internet access [66–68]. Alternatively, models such as “tele-harm reduction” incorporating tele-health and peer support could support linkage to and retention in comprehensive HIV prevention and treatment services following HIVST [69, 70]. Despite these concerns, however, our findings echo a recent scoping review concluding that HIVST is generally preferred in at-risk populations over traditional facility-based HIV testing [71].

Our study had several limitations. First, we recruited service providers in one specific geographic region and socio-political context. However, regarding the transferability (i.e., transportability) of our findings [39], it is important to note that San Diego County shares many characteristics with other jurisdictions across the United States that are impacted by drug use-related HIV transmission (e.g., a geographically-dispersed population and limited public transportation infrastructure, a large and growing population of individuals experiencing unsheltered homelessness who are subjected to frequent, health-harming “street sweeps” and displacement [72], and a rapidly expanding harm reduction service delivery landscape). Second, our relatively brief (~ 45 min) interviews were initially designed to explore service providers’ perspectives on long-acting injectable PrEP delivery to PWUD [33], and we may have missed opportunities to systematically probe about delivering information and supported referrals to local HIV services (for prevention and treatment), which will be critical to maximizing the individual and public health impacts of this approach. Third, we did not utilize a specific implementation science framework to guide the data collection or analysis for this study. Future research could expand upon our findings and more comprehensively investigate a fuller range of implementation determinants by using the Consolidated Framework for Implementation Research, for example [73].

Despite these limitations, we found that health, harm reduction, and social service providers included in our qualitative study generally viewed the peer distribution of HIVST kits among PWUD as promising, appropriate, and feasible. Yet, specialized efforts may be needed to reach the most marginalized individuals and ensure consistent provision of educational information and referral support. Based on SSPs’ trusting relationships with their participants, historical success with secondary distribution of prevention supplies, and ongoing expansion nationally [74], it appears that training PWUD who access SSPs to distribute HIVST kits (along with information on local HIV prevention and treatment services) could activate social influence processes, enhance the credibility of the information shared, establish HIV testing and service engagement as normative, and transform behaviors to support HIV testing and service engagement in PWUD networks.

## Data Availability

The full dataset analyzed for this study are not publicly available due to the sensitive nature of interview questions and disclosure of personal experiences by some participants, but portions of de-identified data may be made available from the corresponding author upon reasonable request.
